# Atrial Antitachycardia Pacing in Complex Congenital Heart Disease: A Case Series

**DOI:** 10.19102/icrm.2018.090304

**Published:** 2018-03-15

**Authors:** Collin C. Kramer, Jennifer R. Maldonado, Mark D. Olson, Jean C. Gingerich, Luis A. Ochoa, Ian H. Law

**Affiliations:** ^1^University of Iowa Carver College of Medicine, Iowa City, IA, USA; ^2^Division of Pediatric Cardiology, University of Iowa Stead Family Children’s Hospital, Iowa City, IA, USA

**Keywords:** Atrial antitachycardia pacing, congenital heart disease, intra-atrial reentrant tachycardia

## Abstract

Among the congenital heart disease (CHD) population, intra-atrial reentrant tachycardia (IART) is a common sequela resulting from anatomical anomalies and surgical scars that significantly increases morbidity and mortality. Atrial antitachycardia pacing (ATP) delivered by atrial antitachycardia devices (ATDs) has been used to treat IART in the CHD population. However, there remains limited data on the safety and efficacy of ATP, as well as on comparisons of its effects amongst different CHD subtypes. The purpose of the current study is to describe the clinical history and ATP efficacy in three patients with unique forms of complex CHD. During this study, a single-center review of three patients with ATDs was performed. One patient with each of the following CHD anomalies was selected for inclusion: systemic left ventricle, systemic right ventricle, and single ventricle. Data collected included ATP success rates, medications in use, direct current (DC) cardioversions, and any complications related to the ATDs. Study findings revealed the patient with a systemic left ventricle had an ATD implanted for approximately 9.5 years, with 695 of 956 (73%) episodes successfully converted. Unsuccessfully treated episodes were generally asymptomatic and self-terminating in this patient. The patient with a systemic right ventricle had an ATD implanted for approximately 16 years, with 333 of 348 (96%) episodes being successfully converted. The patient with a single ventricle had an ATD implanted for approximately 12.5 years, with 404 of 416 (97%) episodes successfully converted. The patients with biventricular physiology were able to forgo DC cardioversion after receiving their ATDs. However, due to medical noncompliance as well as multiple episodes of IART, which presented with 1:1 conduction or low rates, the single-ventricle patient still required DC cardioversions post-ATD implantation. In conclusion, this study’s findings demonstrate that, while ATP can be effective in a wide variety of CHDs, experiences can vary based on individual arrhythmia substrates, cardiac anatomy, and medical compliance. Additionally, challenges remain in IART detection in patients with especially complex CHD anatomies.

## Introduction

Congenital heart disease (CHD) is the most common birth defect in the United States.^1^ Crucial surgical and medical advancements have significantly extended life expectancies; however, cardiac arrhythmias such as intra-atrial reentrant tachycardia (IART) are common in adults with surgically repaired CHD.^2^ IART is defined as any macrorentrant atrial tachycardia that is not necessarily dependent on the cavotricuspid isthmus.^3^ In CHD patients, IART significantly increases morbidity and mortality.^4^ Antiarrhythmic medications are often a suboptimal treatment strategy, and catheter ablations generally yield low success rates in patients with complex CHD.^5–7^

**Figure 1:**
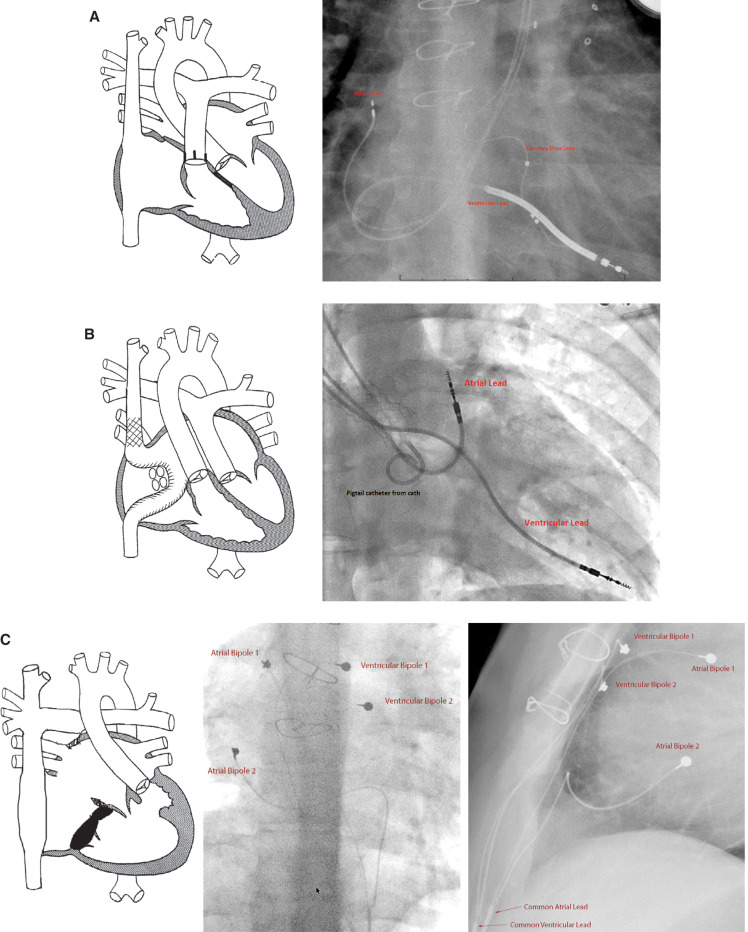
Diagrams for the three cases with adjacent anteroposterior chest radiograph images showing pacemaker lead placement. **A:** Case 1 (systemic left ventricle) involved prior treatment for tetralogy of Fallot. **B:** Case 2 (systemic right ventricle) involved dextro-transposition of the great arteries. **C:** Case 3 (single ventricle) involved double inlet left ventricle, levo-transposition of the great arteries, ventricular septal defect, and ventricular inversion status post-Fontan procedure.

Antitachycardia pacing devices (ATDs) with atrial antitachycardia pacing (ATP) technology were developed primarily for the management of atrial flutter and fibrillation in adults with structurally normal hearts in the mid-1990s. ATDs not only treat IART, but can also prevent atrial tachycardias with atrial preference pacing (APP) and atrial rate stabilization (ARS) modalities. Since their introduction, ATDs have gained popularity as a means of managing sinus node dysfunction and atrioventricular node conduction block in CHD patients. Nevertheless, little literature exists for ATP efficacy in this population, and there are few comparisons in existence of ATP effectiveness between the various CHD subtypes.^8^ After obtaining institutional review board approval, we set out to describe and compare cases in which ATP was successful, using three cases with different cardiac anatomies: one systemic left ventricle, one systemic right ventricle, and one single ventricle, respectively **(Figure 1)**.

## Case presentations

### Case 1: systemic left ventricle

A 22-year-old man with a history of tetralogy of Fallot repair at the age of two years, established care after six years without a pediatric cardiology consultation. On initial presentation, a Holter monitor demonstrated a right bundle branch block with frequent premature ventricular complexes that had varying QRS morphologies. There was significant concern regarding ventricular arrhythmias due to the tetralogy of Fallot repair, right ventricular dilation and dysfunction, ventricular ectopy, poorly contractile left ventricle, and long QRS duration. He was started on enalapril 5 mg twice daily for left ventricular dysfunction and congestive heart failure.

One year later, he presented with pulmonary valve insufficiency and inducible IART during an electrophysiology study. Two months later, at age 24, he underwent concurrent pulmonary valve replacement, right ventricular outflow tract reconstruction, and right atrial MAZE procedures. At discharge, he was prescribed carvedilol 25 mg twice daily and digoxin 0.25 mg twice daily, while his enalapril dosage remained unchanged. However, although his hemodynamic status improved after undergoing this procedure, he continued to experience significant ventricular ectopy. He reported improved exercise tolerance and denied any symptoms relating to arrhythmias, so the medical team opted for careful observation over intervention.

One year later, during an exercise treadmill test, the patient developed 2:1 atrioventricular (AV) conduction, which dropped his ventricular rate to 70 bpm. Given this finding and his aforementioned risk factors for ventricular arrhythmias, he underwent placement of an implantable cardioverter-defibrillator (ICD) with ATP capabilities four months later, specifically a Virtuoso™ DR ICD (Medtronic, Minneapolis, MN, USA) with transvenous right atrial and right ventricular leads. ATP therapies as well as APP and ARS were enabled following the confirmation of lead placement by X-ray one month later.

***Response to ATP***. In the first seven years after device placement, the ATD successfully converted 695 of 956 treated IART episodes (73%) **(Table 1)**. Of note, in one seven-month period, the patient experienced 630 asymptomatic episodes of IART, which the device treated with 70% efficacy. At that time, he was noted to have a long QRS interval (153 ms) and poor left ventricular function; therefore, an upgrade to a biventricular system was considered.

Due to battery depletion, the patient underwent generator replacement with an upgrade to a cardiac resynchronization therapy (CRT) device, with new transvenous lead placed into the left ventricular coronary venous system at age 32 years. His medications were also changed to digoxin 0.125 mg three times weekly, carvedilol 25 mg twice daily, and enalapril 10 mg once daily. One year following the upgrade to the CRT device, he reported improved exercise tolerance, and it was found that his new device had not detected any IART episodes.

Three months later, the right ventricular lead failed secondary to a microfracture. The patient required a three-day hospital stay to remove the defective lead, and he underwent implantation of an entirely new CRT-defibrillator system. For the next two years, his device did not detect any sustained episodes of IART.

### Case 2: systemic right ventricle

This female patient was born with dextrotransposition of the great arteries and a ventricular septal defect. She underwent a Blalock/Hanlon procedure at two weeks of age and a Mustard atrial switch repair at 2.5 years of age. She did well until age 29 years, when she presented to the emergency department (ED) with palpitations, which were diagnosed as IART. She underwent chemical conversion with ibultilide. One year later, she returned to the ED with IART, which was converted with diltiazem. She was prescribed digoxin 0.25 mg once daily and benazepril 5 mg once daily at this time.

Nineteen months later, she returned again to the ED with recurrent IART and underwent direct current (DC) cardioversion. This would be the first of four DC cardioversions that would be required over the next four months. During that time, she was trialed on procainamide and quinidine, neither of which was able to prevent her IART. She subsequently underwent radiofrequency ablation at age 32 years. However, one month later, she presented again to the ED with IART, where she received her fifth DC cardioversion and was sotalol 80 mg twice daily and warfarin.

Six months later, she underwent an electrophysiology study, which found IART and sinus node dysfunction. The IART pathway was ablated and she also underwent stent placement in the superior limb of the systemic baffle in anticipation of a tranvenous pacemaker implant. Three days after this procedure, IART recurred, and she received her sixth DC cardioversion, which resulted in a junctional rhythm.

Two months later, at age 33 years, she underwent the placement of an AT500 pacemaker (Medtronic, Minneapolis, MN, USA) with transvenous leads in the anatomic left atrium and anatomic left (subpulmonic) ventricle. Upon confirmation of the lead positions, ATP therapies were enabled. The device immediately recognized IART, which was successfully converted on the second attempt. APP and ARS were subsequently enabled. Given the immediate success of ATP, her sotalol regimen was discontinued.

***Response to ATP***. Following continued evidence of successful ATP, the patient was able to discontinue warfarin and to halve her digoxin dose to 0.125 mg once daily. The device reached the end of its battery life after converting 31 of 31 (100%) treated IART episodes over five years.

The patient subsequently received an EnRhythm™ generator (Medtronic, Minneapolis, MN, USA), which successfully converted 101 of 105 (96%) of treated IART episodes in the first five years. One IART episode at age 43 years was persistent despite 20 attempted ATP sequences. The episode terminated spontaneously after the patient’s arrival at the ED. She was able to go for three more years with high rates of ATP success and no symptoms. At age 46, however, she experienced a sustained episode of IART (1:1 AV conduction-disabled therapy) and was therefore started on bisoprolol, 2.5 mg daily, and the digoxin was discontinued. At that time, she also underwent ATD generator replacement due to battery depletion, receiving an Advisa pacing system (Medtronic, Minneapolis, MN, USA). Six months after implantation of this device, she complained of pain at the incision site and it was observed that there was thinning of the subcutaneous fat. Therefore, a pocket revision was performed. In the two-year period following her receiving this third ATD, there were no documented episodes of sustained IART. Over the approximately 16 years that this patient had an ATD implanted, 333 of 348 IART episodes (96%) were successfully converted by ATP **(Table 1)**.

### Case 3: single ventricle morphology

The final patient included in this study had a history of double inlet left ventricle and levotransposition of the great arteries. He had undergone pulmonary banding at six weeks of age and an atriopulmonary Fontan procedure at age eight years. He presented at age 14 years following a syncopal episode and a subsequent episode of near-syncope. One week later, he again experienced near-syncope, with a heart rate of 35 bpm. He was hospitalized and a Holter monitor found no episodes of arrhythmia, but junctional rhythms with bradycardia (40 bpm) were noted. Cardiac catheterization at that time found adequate Fontan pressures (mean: 12 mmHg).

The patient was lost to follow-up until returning to the clinic seven years later at the age of 21 years. At that time, he had experienced two episodes of symptomatic IART, neither of which was associated with syncope. Over the subsequent six months, he experienced two additional episodes of IART requiring cardioversions. One year later, he again lost consciousness and required a DC cardioversion for IART. Following this episode, he was prescribed atenolol 25 mg once daily and underwent an electrophysiology study, which detected an inducible IART pathway that was subsequently ablated in two locations.

One week later, symptomatic IART recurred and he underwent another DC cardioversion. His daily atenolol dose was increased to 50 mg. Several days after, IART recurred and required DC cardioversion again. The patient had been noncompliant with the increased atenolol dose during that time. He was started on sotalol 80 mg twice daily. An attempt was made to increase the sotalol to 120 mg twice daily; however, the patient developed nausea and headaches. IART recurred two weeks later and he required a fifth DC cardioversion. Following this recurrence, his sotalol was successfully increased to 120 mg twice daily, and digoxin 0.125 mg once daily was added.

He underwent a second electrophysiology study several weeks later and two IART circuits were identified and ablated; however, five hours later, IART recurred. The arrhythmia was converted chemically and he was prescribed amiodarone 500 mg once daily.

Several months later, he underwent concurrent lateral tunnel Fontan conversion, right-sided maze, right atrial reduction plasty, intra-atrial septectomy, and temporary epicardial pacemaker lead placement on the anatomic right ventricle and atrium. Hours after the surgery, the patient developed IART, which was successfully overdrive-paced. Following this episode, the decision was made to implant a permanent pacemaker-generator. Several days later, he received an AT500 pacemaker (Medtronic, Minneapolis, MN, USA) with ATP and APP enabled, and his daily amiodarone dose was reduced to 200 mg. At the time of his pacemaker implant, he was taken off digoxin.

***Response to ATP***. One-month after receiving his ATD, it failed to convert IART. It was suspected that the ATD was unable to detect an IART circuit that was slower than previously documented arrhythmias. Two months later, his device again failed to treat IART due to 1:1 AV conduction, which disabled the ATP therapy. Therefore, his ventricular lead was capped and the ATD was reprogrammed to prevent disabling of the ATP. He also discontinued the amiodarone and was started on digoxin 0.125 mg daily.

Following these changes, the ATD converted 347 of 357 episodes (97%) of IART. Nevertheless, he required frequent visits to the ED for cardioversions or manual overdrive pacing secondary to sustained IART that was resistant to ATP or not detected by his device. Changing from digoxin to sotalol 80 mg twice daily was not successful in preventing IART.

At the age of 30 years, he underwent a generator change due to battery depletion and received an EnRhythm™ device (Medtronic, Minneapolis, MN, USA). He continued to have high success rates of ATD efficacy for detected episodes; however, several instances of IART went undetected, with a number of them requiring manual overdrive pacing and three needing cardioversion, respectively. At that time, it was suspected that the detection errors were due to far-field oversensing. Additionally, the patient continued to have issues with medication compliance. Of the 46 episodes of ATP that were detected and treated by the second device, 44 (96%) of them were converted.

At the age of 35 years, he again underwent a generator change due to battery depletion and received an Advisa pacemaker (Medtronic, Minneapolis, MN, USA). In the first year after he received this third device, 13 of the 13 (100%) episodes he experienced were detected and treated successfully with ATP. In this time period, there were no episodes of incessant IART requiring cardioversion. In his approximately 13 years total with an ATD, there were 416 detected and treated episodes, of which 404 (97%) were converted by ATP **(Table 1)**.

## Discussion

We have determined that ATP therapy can be effective in patients with vastly different cardiac anatomies. This is in contrast with ATP therapy in the structurally normal heart for the management of complex atrial flutter and atrial fibrillation, where efficacy has been more limited.^9,10^ That being said, efficacy in the CHD population varies based on individual arrhythmia substrates, cardiac anatomy, and medical compliance.

The patient in case 1 experienced the lowest overall rate of ATP success (73%) but also had the most benign treatment course, experiencing no symptomatic IART nor requiring any cardioversions. In case 2, the patient enjoyed a high ATP success rate of 96%. She experienced two episodes of IART, which were resistant to ATP, though neither required cardioversion. Having required six DC cardioversions and anticoagulation prior to ATD implantation, her IART burden was significantly reduced. Of note, while cases 1 and 2 enjoyed high rates of success, both required additional procedures related to ATD system issues.

Case 3 proved to be particularly problematic with regards to ATP. Although this single-ventricle patient enjoyed the highest overall rate of ATP success (97%), the episodes that went undetected or that were untreatable proved burdensome, requiring frequent cardioversions or manual overdrive pacing. It’s unclear if empiric removal of the ventricular lead aided in IART detection, but this case did demonstrate the safety of the technique. It should also be noted that this particular case occurred in a setting of medication noncompliance.

Our findings advocate for the continued use of ATDs in the management of IART in the CHD population and highlight that each case must be evaluated on an individual basis. Further research and development into ATP modifications for use with atypical cardiac anatomies may prove beneficial.

## Figures and Tables

**Table 1:** ATP Episodes and Success Rates for Each Case

Case	Number of ATP Episodes Treated	Number of ATP Episodes Successfully Converted	ATP Success
Systemic left ventricle (case 1)	956	695	73%
Systemic right ventricle (case 2)	348	333	96%
Single ventricle (case 3)	416	404	97%

ATP: antitachycardia pacing.
